# *FOXN1*: A Master Regulator Gene of Thymic Epithelial Development Program

**DOI:** 10.3389/fimmu.2013.00187

**Published:** 2013-07-12

**Authors:** Rosa Romano, Loredana Palamaro, Anna Fusco, Giuliana Giardino, Vera Gallo, Luigi Del Vecchio, Claudio Pignata

**Affiliations:** ^1^Department of Translational Medical Sciences, “Federico II” University, Naples, Italy; ^2^Department of Biochemistry and Medical Biotechnology, CEINGE Institute, “Federico II” University, Naples, Italy

**Keywords:** Foxn1 gene, TECs, thymus gland, immunodeficiency, Nude/SCID

## Abstract

T cell ontogeny is a sophisticated process, which takes place within the thymus through a series of well-defined discrete stages. The process requires a proper lympho-stromal interaction. In particular, cortical and medullary thymic epithelial cells (cTECs, mTECs) drive T cell differentiation, education, and selection processes, while the thymocyte-dependent signals allow thymic epithelial cells (TECs) to maturate and provide an appropriate thymic microenvironment. Alterations in genes implicated in thymus organogenesis, including *Tbx1*, *Pax1*, *Pax3*, *Pax9*, *Hoxa3*, *Eya1*, and *Six1*, affect this well-orchestrated process, leading to disruption of thymic architecture. Of note, in both human and mice, the primordial TECs are yet unable to fully support T cell development and only after the transcriptional activation of the *Forkhead-box n1* (*FOXN1*) gene in the thymic epithelium this essential function is acquired. *FOXN1* is a master regulator in the TEC lineage specification in that it down-stream promotes transcription of genes, which, in turn, regulate TECs differentiation. In particular, *FOXN1* mainly regulates TEC patterning in the fetal stage and TEC homeostasis in the post-natal thymus. An inborn null mutation in *FOXN1* leads to Nude/severe combined immunodeficiency (SCID) phenotype in mouse, rat, and humans. In *Foxn1*^−/−^ nude animals, initial formation of the primordial organ is arrested and the primordium is not colonized by hematopoietic precursors, causing a severe primary T cell immunodeficiency. In humans, the Nude/SCID phenotype is characterized by congenital alopecia of the scalp, eyebrows, and eyelashes, nail dystrophy, and a severe T cell immunodeficiency, inherited as an autosomal recessive disorder. Aim of this review is to summarize all the scientific information so far available to better characterize the pivotal role of the master regulator FOXN1 transcription factor in the TEC lineage specifications and functionality.

## Introduction

The thymus is the primary lymphoid organ with the unique function to produce and to maintain the pool of mature and functional T cells. This process is strictly dependent on specialized functions of thymic stromal cells (TSCs) and requires the thymus peculiar tridimensional (3D) architecture, which allows a proper intercellular cross talk (1). For a long time, the difficulty in the isolation and characterization of the thymic cellular components has limited studies on the peculiar role of individual stromal components. Novel experimental tools, including stromal cell isolation by phenotype-based cell sorting (2), dissociation and reaggregation of stromal cell subsets (3, 4), or global gene expression analysis and the evaluation of the pattern of self-antigen expression within the individual thymic epithelial cells (TECs) subset (5), allowed to acquire important knowledge on the cellular and molecular basis of thymus organogenesis and TECs functionality.

The recent discovery of disease models associated to genetic alterations of molecules implicated in thymus specification and TECs differentiation, provided new and conclusive insights regarding the pathways, the genes, and the molecular mechanism governing these processes and stromal functionality.

## The Thymus Architecture: Requirement of a 3D Structure for a Proper Lympho-Epithelial Crosstalk

The thymus provides the microenvironment essential for the development of T cells. T cell progenitors originate in the bone marrow, enter into the thymus (6, 7) and, through a series of well defined and coordinated developmental stages, differentiate, undergo selection process, and mature into functional T cells. The steps in this process are tightly regulated through a complex network of transcriptional events, specific receptor-ligand interactions, and sensitization to trophic factors, which mediate the homing, proliferation, survival, and differentiation of developing T cells (1, 8, 9).

The thymus is organized in two lobes, which are already present in mice at 21 days of thymic organogenesis and is completely organized at 1 month of post-natal life. The lobes are divided in three areas: a cortical and the dark cortical area, with a high number of lymphoid cells and epithelial cells, cortical thymic epithelial cells (cTECs); a light medullary area with a low number of mature T cells, named medullary TECs (mTECs), Hassall’s bodies (HB), macrophages, dendritic cells (DCs), B lymphocytes, and rarely myoid cells. Eventually, there is a transitional area, named cortico-medullary junction (CMJ), characterized by abundant blood vessels (10).

The unique function of the thymus in the establishment and maintenance of the T cell pool is intimately linked to this peculiar thymus architecture and to the specialized functions of the TSCs.

## Lympho-Epithelial Cross-Talk Required for Thymocyte and TECs Differentiation

An important feature of the thymic microenvironment is its 3D organization, consisting of an ordered architecture of TSCs, that represents a heterogeneous mixture of distinct cell types, including cTECs, mTECs, fibroblasts, endothelial cells, DCs, and macrophages (11). Among these stromal elements, TECs are the most abundant cell types, which form a delicate 3D cellular network spanning throughout both the thymic cortex and the medulla. The requirement for the 3D-supporting stroma appears to be unique to the T cell development, as the *in vitro* differentiation program of other hematopoietic lineages, including B and NK cells, does not require a 3D structure (12).

Thymocyte development is not a cell-autonomous process, and the transition to the next stage in development relies on the proper interaction of HSCs with thymic stroma. The 3D configuration of the thymus maximizes this interaction, allowing intercellular cross-talk integral to the development of both T cells and TSCs (13). Paralleling the T cell precursor proliferation and differentiation program, immature TECs undergo a developmental sequence, resulting in the establishment of mature cTECs and mTECs organized in this 3D network. Several studies on mutant mice with an abnormal organization of thymic epithelium substantiated the concept that a reciprocal signaling between thymocytes and TSCs is required, not only for the production of mature T cells but also for the development and organization of the thymic microenvironment in a bi-directional fashion (14, 15). Mice showing a blockage of the T cell development process, in the absence of T cell receptor (TCR)-expressing cells, have a defective organization of the thymic medulla, as well (16, 17). Of note, under this condition, thymic medullary organization can be restored by the addition of mature T cells, which follows stem cell transplantation (17, 18). In adult CD3etg26 mice, lacking intra-thymic T cell precursors, a severe alteration of the cortical thymic architecture has been documented (19), even though a restoration of the architecture and TEC development in these mice can occur. Recently, the injection of either fetal or adult T-committed precursors into adult CD3etg26 mice leads to the reconstitution of thymic microenvironment, as indicated by thymocyte differentiation, organization of functional cortical and medullary areas, and generation of Foxp3^+^ T_reg_ and Aire^+^ mTECs (20). These data suggest that adult TECs maintain the receptivity to cross talk with thymocytes despite a prolonged absence of T cell precursors. Moreover, the absence of both thymocytes and of the 3D framework may result in changes of the keratin genes expression, thus inducing the cTECs and mTECs to undergo a de-differentiation process and to reacquire the precursor K5^+^K8^+^ cellular phenotype. Taken together, these findings suggest that signals from early CD4^–^CD8^–^ DN T cell precursors and/or their immediate progeny provide necessary signals to promote the formation of the thymic cortex, while, later in ontogeny, the differentiation of TECs into a medullary phenotype are clearly dependent on the presence of CD4^+^CD8^−^ and CD4^−^CD8^+^ single positive (SP) thymocytes (21–23). However, the precise molecular nature of the signals provided by developing thymocytes, which lead to the generation of the thymic stromal compartment are still incompletely defined.

Eventually, a better understanding of the developmental process through which a normal thymus structure is built, is essential for a better comprehension of the intimate mechanisms which take place within the thymus to promote the T cell development *in vivo*. This knowledge may also be useful in designing future therapeutic strategies, as alterations of the thymus structure and function may result in serious health consequences, including immunodeficiency or autoimmunity.

## mTECS and cTECS are Specialized Cells Playing a Different Role in the T Cell Education Process

T cell ontogeny is a sophisticated process, which takes place through discrete stages during which developing thymocytes dynamically relocate in different thymic areas, following a cortico-medullary gradient.

**Figure 1:**
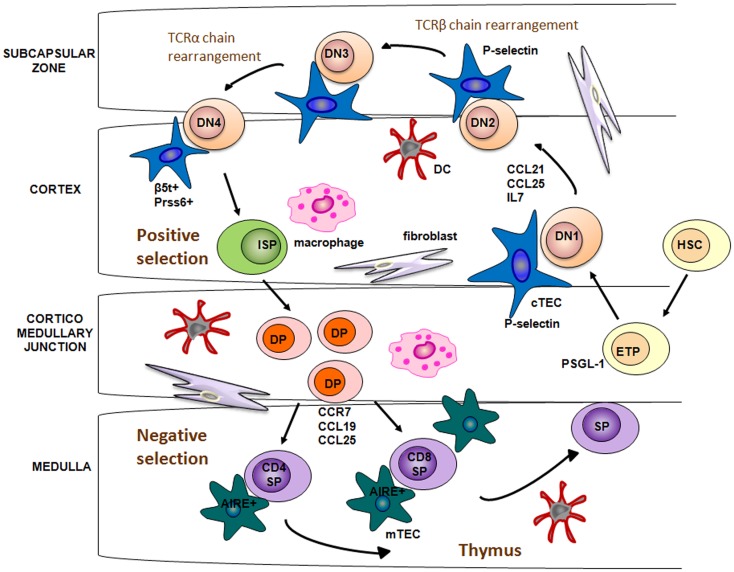
**Lympho-stromal interactions and T cell development**. Bone marrow HSCs enter into the thymus through CMJ, a process mediated by CCL12 and CCL25 in the embryonic thymus and by the interaction between P-selectin and its cognate ligand PSGL-1 in adult thymus. Stimulation by IL-7 allows the relocation of DN thymocytes from the cortex to the subcapsular region. DP thymocytes bearing TCR and capable of binding to self-MHC ligands are positively selected. This process is regulated by Pssr6 and b5t, which are expressed in cTECs. Developing thymocytes are relocated from cortex to the medulla by chemotactic attraction between CCR7 and the ligands CCL19/CCL21, expressed on the mTECs. Into medulla, self-reactive thymocytes are deleted through the negative selection, a process mediated by dendritic cells and Aire-expressing mTECs.

The initial colonization of the thymus anlagen by migrant lymphoid progenitors occurs at an early stage, embryonic day 11.5 (E11.5) in mice and 8 week of gestation in humans (24, 25). Studies documented that chemokines CC ligand (CCL)21 and CCL25 play a major role in the early stage of fetal thymus colonization (26, 27). Indeed, mice deficient for these chemokines or for the cognate receptors, showed a significant reduction in the number of thymocytes compared to normal mice (28). In post-natal thymus, lymphoid progenitor cells through their cell surface adhesion molecules, such as platelet-selectin glycoprotein ligand 1, interact with P-selectin, expressed on the TECs, and thanks to this interaction they are allowed to migrate from the blood into the thymic parenchyma, in correspondence of the area around the CMJ [Figure 1; (29)].

Entered thymocytes started to intensely proliferate and to acquire T cell hallmarks. In this phase, T cell proliferation and differentiation are triggered by a potent combination of signals provided by cTECs. Delta-like 4 (DL4), which is an essential, non-redundant ligand for Notch1 during thymic T cell development, and IL-7 are critically involved in the activation of signaling pathways, leading to the proliferation and migration of thymocytes (30–32). In particular, these intra-thymic ligands induce the development of DN CD25^+^ cells, which migrate toward the subcapsular region of thymic cortex (33). Several chemokine receptors have been suggested to guide the migration of immature thymocytes, such as CXCR4, CCR7, and CCR9 [Figure 1; (34)]. In the thymic cortex DN thymocytes begin V(D)J rearrangement of their TCRβ gene. Successfully rearranged TCRβ protein, assembled with the pre-TCRα chains, forms the pre-TCR complex. Membrane expression of pre-TCR complex, along with the Delta-Notch interaction, provides the signal necessary to induce the expression of the co-receptors CD4 and CD8, as well as V-J rearrangement of the TCRα genomic region. Subsequently, DP thymocytes with a functional TCR-αβ receptor are generated [Figure 1; (35)].

Thymic cortex is also the area where takes place the positive selection of DP thymocytes. Positive selection is the process by which developing thymocytes, that recognize and bind with mild avidity peptide-major histocompatibility complex on cTECs surface, get a rescue signal through their TCR and are allowed to further maturate to the CD4^+^CD8^−^ or CD4^−^CD8^+^ SP stage. Only a small fraction (1–5%) of DP cells survive to positive selection. By contrast, the majority of DP cells, that bind with too low affinity to MHC complex, are programed to undergo death by neglect (36, 37).

Cortical thymic epithelial cells have a crucial role in the positive selection process of T cells within thymus cortex (38). Recent studies have found that cTECs exclusively express a specific form of proteasome, referred as thymoproteasome, which contains a peculiar catalytic subunit, the β5-thymus (β5t) (39). β5t subunits exhibit an unique peptidase activity, compared to other β5 subunits found in common immunoproteasome, which leads to the production of a set of self-peptides with a high affinity for class I MHC molecules (40). Moreover, β5t-deficient mice show a severe decrease in the number of CD8^+^ SP thymocytes, but no alteration in the CD4^+^ number or in the thymic architecture. In addition, the small fraction of CD8^+^ T cells, positively selected by β5t-deficient cTECs, show altered immune responses toward several stimuli. Taken together these results suggest that the thymoproteasome is essential for the production of self antigens involved in the positive selection of functional CD4^−^CD8^+^ T cells (41).

As for the positive selection of CD4^+^ T cells, two other proteins predominantly expressed in cTECs, the lysosomal protease Prss16 and Cathepsin L, have been demonstrated to be essential to generate an immunocompetent repertoire of CD4^+^CD8^−^ T cells [Figure 1; (42, 43)].

TCR engagement by peptide-MHC complex also triggers the expression of the chemokine receptor CCR7 in positively selected thymocytes. Thanks to the chemotactic attraction between CCR7 and its ligands, CCL19 and CCL21, expressed on the mTECs, developing thymocytes are relocated from cortex to the medulla [Figure 1; (44, 45)].

In order to create a repertoire of mature T cells able to recognize foreign antigens and, at the meantime, to ignore self antigens, SP thymocytes have to undergo the negative selection process in the thymic medulla. Both mTECs and DCs, play a pivotal role in this last stage of thymocyte development, which is critical to establish the central tolerance and, eventually, to prevent autoimmunity. In contrast to cTECs, mTECs are characterized by a high expression of clustered tissue-restricted autoantigens (TSAs), the so called promiscuous gene expression (46). To date, the autoimmune regulator (AIRE) transcription factor represents the only molecule, so far identified, which contributes to the mTECs function and, in particular, to the molecular regulation of the promiscuous gene expression [Figure 1; (47)]. However, not all TSAs are regulated in an AIRE-dependent manner, suggesting that other molecular mechanisms, such as epigenetic mechanisms, may be involved in mTECs function regulation. TSAs associated with class II MHC molecules are presented directly by mTECs or indirectly by DCs to developing thymocytes (48). T cells which recognize with a high avidity self antigens are deleted. Remarkably, only a few number of mTECs express a given TSAs (about 50–500 per thymus), and lead to apoptosis by negative selection of a few thymocytes (37, 49, 50). A possible explanation is that the high motility of thymocytes within the thymic medulla during a period of 4–5 days, allows each of them to interact with mTECs (51). DCs play a similar role in the negative selection process. They are attracted in the thymic medulla by the chemochine XCL1 (lymphotactin), produced by mTECs in an AIRE-dependent manner. Differently from mTECs, DCs are not able to produce TSAs and the TSAs expressed mostly derive from the phagocytosis of apoptotic mTECs (52, 53). mTECs and DCs not only contribute to the establishment of central tolerance through the deletion of self-reactive T cells, but, also, through the generation of regulatory T cells (T_regs_) (54, 55, 65, 153), which act in the periphery by suppressing autoreactive T cells, which have escaped to the process of the central tolerance.

A body of evidence documents that the expression of an autoreactive TCR leads to the entry of the thymocyte into the T_reg_ lineage. T_regs_, that are about 5–10% of peripheral T cells CD4^+^, constitutively express the CD25 molecule and share several immunological features, in humans and mice (56, 57). These cells specifically express the transcription factor FOXP3 (Foxp3 in mice) that plays a pivotal role in T_regs_ differentiation and function (58). The Foxp3 promoter region and the conserved non-coding sequence 2 (CNS2) (known as TSDR, the T_reg_-specific-demethylated-region) are fully methylated in immature thymocytes (59, 155). At the beginning of T_reg_ development, an appropriate TCR/CD28 signal is needed to make available the *Foxp3* promoter through shift of the Protein Inhibitors of Activated STAT 1 (PIAS1), a signal cascade, which results in the NF-κB-mediated transcription of genes playing a role in T_reg_ differentiation (60, 61).

## Thymic Formation: New Insights in Epithelial Lineages Specification

In the mouse, mTECs and cTECs originated from the third pharyngeal pouch endoderm and the thymus anlage are located next to that of the parathyroid. The expression of Forkhead-box transcription factor n1 (Foxn1) approximately at E11.5 is crucial for the subsequent epithelial differentiation, since in its absence, the colonization of the anlage by T cell progenitors from the bone marrow fails (62) and the subsequent T cell development and TECs formation is aborted, resulting in a severe immunodeficiency (63, 64, 154, 66).

The maturation process of TECs during thymic organogenesis could be divided in two genetic phases. The first stage is independent from the *Foxn1* expression and consists in the induction and outgrowth of the thymic epithelial anlage from the third pharyngeal pouch, through the expression of genes including the *Eya1* and *Six* (67), *Hoxa3* (68), and *Tbx1* (69, 70). During the second genetic phase, epithelial patterning and differentiation take place and the *Foxn1* expression drives the immature epithelial cells to differentiate into functional cTECs and mTECs (71).

## FOXN1-Indipendent Genetic Stage of TEC Differentiation

In the first phase of the thymus organogenesis an interaction between epithelial and mesenchymal cells occurs, while at the later phase lympho-epithelial interaction predominates (72). In mice, at about E10.5 the mesenchymal cells are able to respond to the endodermic signals, which induce the development of the primordial thymic epithelium (73, 74). Subsequently, at about E12.5, the thymic rudiment is colonized by progenitors come from the fetal liver, thus resulting in a tight epithelial-thymocyte interaction within the mesenchymal derived capsule. This thymic rudiment contains the EpCam^+^Plet1^+^ epithelial population (72, 75), which includes a common thymic epithelial precursor (TEPC), from which both cTECs and mTECs will be subsequently generated (72, 76).

Through studies on animal models carrying molecular alterations of distinct genes, the key role of several transcription factors involved in the thymus organogenesis and TEC-sublineage specification process, have thus far been identified (77). In particular, several genes, including *Tbx1* (69, 70), *Pax1*, *Pax3*, *Pax9* (78–80), *Hoxa3* (68), *Eya1*, and *Six1* (67) have been shown to play a central role in the thymus ontogeny. Indeed, their molecular alteration affects this well-orchestrated process, leading to disruption of the thymic architecture. Abnormalities of the paired box (Pax) family transcription factors Pax1 or Pax9 result in a blockage of the thymus organogenesis (79, 81). Mutations in the Hox transcription factor family member, Hoxa3, expressed on both thymic epithelium and mesenchymal cells, result in athymia (68). Furthermore, the homozygous loss of *Tbx1*, related to the DiGeorge syndrome phenotype, leads to thymic a/hypoplasia in humans (69, 82), while mice heterozygous for a null allele of *Tbx1* show a mild phenotype without thymus anomalies (83). Therefore, the expression of *Tbx1* both in the pharyngeal core mesoderm and in the pharyngeal endoderm is required for a proper thymus development. However, it remains to be elucidated whether the expression of *Tbx1* in the TECs occurs and whether the gene participates in the TECs development (4).

## FOXN1-Dependent Genetic Stage of TEC Differentiation

In both humans and mice, the primordial TECs are yet unable to fully support T cell development and only after the transcriptional activation of the *FOXN1* gene in the thymic epithelium this essential function is acquired. *FOXN1* is a master regulator in the TEC lineage development in that it promotes down-stream the transcription of genes implicated in the thymus organogenesis and TECs full differentiation.

Forkhead-box n1 transcription factor belongs to the FOX transcription factor family implicated in a variety of biochemical and cellular processes, including development, metabolism, aging, and cancer (84, 85). During the post-natal life, *Foxn1* is selectively expressed only in thymic and skin epithelia, where it regulates the expression of several molecular targets to maintain the balance between growth and differentiation (86, 87). The signals required for *FOXN1* expression, and its activity, are still unclear, even though the wingless (Wnt) proteins (88) and bone morphogenetic protein (BMP) signaling have been shown to regulate *FOXN1* expression (89). Even though the complete pattern of *FOXN1* expression over the time and its role are not yet completely defined, studies on mouse and human model of gene alterations enormously helped unravel important issues on its role. Mutations in *Foxn1* gene lead to alymphoid cystic thymic dysgenesis due to a defective TECs differentiation process (63, 90). In both mice and humans *FOXN1* abnormalities lead to a hairless phenotype (87, 154).

In the *Foxn1*-dependent step of thymus organogenesis, precursor epithelial cells differentiate into mature and functional cTECs and mTECs from the same bi-potential TEC progenitor (4, 72, 76). It has been reported, that *Foxn1* is differentially expressed during the TE-lineage specification, since it is expressed in all TECs during the pre-natal life, but not in all TECs postnatally, indicating that the gene is highly developmentally regulated. There is a body of evidence documenting different effects of *Foxn1* expression in mTEC and cTECs. Particularly, studies on K5- and K18-CreERT-mediated *Foxn1*-deleted mouse models suggested that during the post-natal life, the loss of *Foxn1* affected mTECs, characterized by the expression of K5 and K14 keratins type. Conversely, the loss of *Foxn1* did not affect cTECs, which express the keratins K8 and K18 (91, 92). Taken together, these data suggest that cTECs and mTECs are not equally *Foxn1*-dependent in the post-natal life.

Recent reports highlighted a central role for *Foxn1* in TECs homeostasis in the adult thymus and its necessary role for the functionality and survival of adult TEC progenitors (92), expressing K5^+^ and K14^+^ markers. This role in adult thymus seems to be exerted in cooperation with other stem cell-related genes, such as *p63*. Of note, the transcription factor p63, encoding for multiple isoforms (93), plays a pivotal for the development of stratified epithelia of several tissues, such as epidermis, breast, prostate, and thymus (94). In the thymus, the p63 protein drives the proliferation of epithelial progenitor cells (94, 95). Therefore, it has been hypothesized that *p63* and *Foxn1* could act synergistically through the formation of a *p63*-*Foxn1* regulatory axis aimed at regulating TECs homeostasis. However, the molecular mechanism through which the proliferation regulator *p63* and differentiation regulator *Foxn1* collaborate in this axis are still unclear.

## FOXN1-Mediated Gene Expression for TEC Differentiation

*Forkhead-box n1* is directly or indirectly implicated in the transcriptional regulation of a panel of genes involved in thymus development and function.

**Figure 2:**
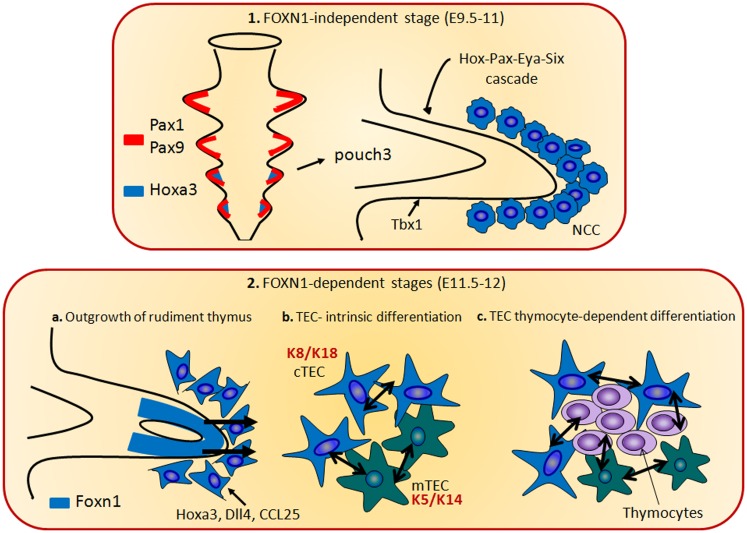
**The thymus organogenesis**. The thymus organogenesis is characterized by two genetic phases. The first stage is independent from the *Foxn1* expression (1) and consists in the induction and outgrowth of the thymic epithelial anlage from the third pharyngeal pouch, through the expression of genes including the *Eya1*, *Six*, *Hoxa3*, and *Tbx1*. *Hoxa3* and *Eya1* are also required in neural crest cells (NCCs). In the phase 2, *Foxn1* regulates the expression of *CCL25*, *Dll4*, and *Hoxa3*, necessary for the thymocytes and TECs differentiation. During this step, cTECs (expressing K8 and K18 keratin type) and mTECs (expressing K5 and K14 keratin type) originate from the same bi-potential TEC progenitor. The crosstalk between TECs and developing thymocytes is required to generate fully mature TECs and functional T cells.

*Pax1* is a key regulator of TEC differentiation/survival balance. *Pax1* is expressed in the third pharyngeal pouch from E9.5 during the thymus ontogeny, while in the post-natal thymus only in cTEC (96). Even though the regulation of *Pax1* is still unclear, from E11.0 its expression requires *Hoxa3* (68). Of note, the loss of *Hoxa3* impairs the intrinsic ability of the neural crest cell population to differentiate and/or to lead to the differentiation of the tissues of pharyngeal arch and pouch. Indeed, in *Hoxa3* mutant mice the thymus is absent and thyroid hypoplasia has been documented (68). Moreover, the first step of thymus development is the expansion of mesenchymal neural crest in the posterior part of the third pharyngeal pouch. Prior to this event, in the *Hoxa3* mutant embryos a marked reduction in *Pax1* expression has been shown. Similarly, *Pax1* mutant mice also show thymic hypoplasia, suggesting a role for *Hoxa3* in maintaining *Pax1* expression in these cells (68). In the thymic primordium, *Pax1* expression is under the control of *Foxn1* (71). This finding indicates that *Foxn1* and *Hoxa3* are both involved in the network of molecular signals that regulates *Pax1* expression, thus demonstrating the existence of a molecular and/or functional interaction between *Hoxa3* and *Foxn1* [Figure 2; (71)]. In keeping with this, Hoxa3^+/−^Pax1^−/−^ compound mutant mice display a few phenotypic hallmarks of the Foxn1^R/R^ mouse model, which expresses low-dose of *Foxn1*, such as hypomorphic post-natal thymus, and reduced levels of MHC class II expression on the TECs surface (80). These data suggest two alternative hypothesis: *Hoxa3* may regulate *Foxn1*, which, in turn, regulates *Pax1* expression in the thymic primordium, in a *Foxn1*-dependent manner, or *Hoxa3* and *Foxn1* induce *Pax1* expression in the third pharyngeal pouch and in early thymus primordium.

It has also been shown that *Foxn1* regulates the expression of *CCL25* and *Dll4* (Figure 2). These genes play a pivotal role in the thymocyte development, since *CCL25* regulates the colonization of the fetal thymus (97), while the Notch ligand *Dll4* is involved in the commitment of hematopoietic progenitors to the T cell lineage (30). In both early fetal TEC and in the post-natal thymus, *Dll4* expression is directly related to the *Foxn1* expression (71). Furthermore, these molecules are absent in the *Foxn1* null thymus, even though there is evidence indicating that their expression may occur in a *Foxn1*-independent manner in TECs (98, 99). Eventually, in a recent report it has been shown that *Foxn1* is upstream of dll4a and ccl25a expression in *medaka fish*, thus confirming the relationship with this transcription factor (100).

## The Human Nude/SCID Phenotype: A Model of Thymic Microenvironment Disruption and Failure of the T Cell Development

The Nude/severe combined immunodeficiency (SCID) phenotype represents the prototype of thymic architecture disruption due to alterations of the *FOXN1*, which is the master regulator of TE-lineage specification (71).

In humans, as in mice and rats, mutations in the “nude” *Foxn1* gene induce the hairless phenotype, associated with a rudimentary thymus gland (T cell related primary immunodeficiency). The human Nude/SCID phenotype (MIM 601705; Pignata Guarino Syndrome) was first identified in 1996, after more than 30 years from the initial mouse description, in two sisters originated from a small community with a high grade of inbreeding, who showed congenital alopecia of the scalp, eyebrows, and eyelashes, nail dystrophy, and a severe T cell immunodeficiency, inherited as an autosomal recessive disorder (154). This phenotype was associated with a C792T transition in the *FOXN1* gene, which resulted in the nonsense mutation R255X in the exon 4 (formerly exon 5), with a complete absence of a functional protein similar to the previously described rat and mouse *Foxn1* mutations (101–103).

In the absence of *Foxn1* expression, thymic development is halted at a rudimentary stage. As a consequence, in the affected patients the thymic lobe is still present but intra-thymic lymphopoiesis is completely blocked (63, 104) leading to severe primary T cell immunodeficiency (105–107) and to death in early childhood from severe infections (105, 108–112, 154). *Foxn1* is also involved in morphogenesis and maintenance of the 3D thymic micro-structure, which is necessary for a fully functional thymus (113, 114). In fact, evidence is available that in an *in vitro* 2D culture system consisting of a monolayer of mouse bone marrow stromal OP9 cells it is possible to generate mature T cells, only if these cells are transduced with the Notch ligand Delta-like 1 (OP9-DL1) (115, 116), whose pathway exerts a pivotal and necessary role in promoting the induction of T cell-lineage commitment (117–119). Of note, in all these co-culture systems, the stromal cells are enforced to overexpress Notch ligands, and their expression by TECs seems to be maintained only in a 3D thymus structure (120). In human Nude/SCID, the T cell defect is characterized by the absence of proliferative response to the common mitogens and a severe blockage of the T cell differentiation (154). Recent studies revealed the presence of some circulating T cells of non-maternal origin in patients carrying alterations of *FOXN1* gene. These cells have been shown to be predominantly double-negative αβ T cells (CD3^+^CD4^−^CD8^−^, DN) and to exhibit a regulatory like T cell phenotype (FoxP3^+^). This finding raised important issues regarding the site of differentiation of these cells. One hypothesis is the persistence of a thymic rudiment, which allows a partial T cell development (109). Alternatively, a T cell differentiation, even though partial and ineffective to result in a productive immunity, could occur at an extra-thymic site. In both pre-natal and post-natal life, the TCRBV spectratype repertoire in Nude/SCID patients is oligoclonal, thus confirming the immaturity of the process and, at the same time, that developmental events do take place at some extent (111, 112).

For many years, the human counterpart of the nude mouse phenotype has been erroneously considered the DiGeorge syndrome, which occurs spontaneously and is mainly characterized by thymic hypo/aplasia and a mild T cell defect. However, several lines of evidence argue against the analogy between these two disorders. In fact, the DiGeorge syndrome is often associated with neonatal tetany and major anomalies of great vessels. These defects are due to malformation of the parathyroid and heart, derived from a major embryologic defect in the third and fourth pharyngeal pouch from which the thymus primordium emerges. In addition, in this syndrome hairlessness is missing and gross abnormalities of skin annexa are not found. Children with DiGeorge syndrome may also have lymphopenia, with a mild reduction of T cells, that are however usually responsive to common mitogens.

In Nude/SCID patients, skin is tighter than usual and is characterized by basal hyperplasia and dysmaturity. Alopecia is primitive in nature, in that it can be observed at birth and persists after bone marrow transplantation, thus ruling out the acquired nature of the disorder. In keeping with this, in athymic mice, completely lacking body hair, restoration of the thymus did not lead to hair growth, indicating a direct participation of FOXN1 to hair follicle development (87). The most frequent phenotypic alteration affecting the nails is koilonychia (“spoon nail”), characterized by a concave surface and raised edges of the nail plate, associated with significant thinning of the plate itself; canaliform dystrophy and a transverse groove of the nail plate (Beau line) may also be observed (121). However, the most specific phenotypic alteration is leukonychia, characterized by a typical arciform pattern resembling a half-moon and involving the proximal part of the nail plate. These alterations of digits and nails were also reported in a few strains of nude mice. Of note, nail dystrophy has also been observed in heterozygous subjects carrying *FOXN1* alterations (121). *FOXN1* is known to be selectively expressed in the nail matrix, where the nail plate originates, thus confirming that this transcription factor is involved in the maturation process of nails and suggesting nail dystrophy as an indicative sign of heterozygosity for this molecular alteration (121).

Autoptical study of a fetus homozygous for R255X mutation revealed multiple-site neural tube defects, including anencephaly and spina bifida. This finding may help explaining the high rate of mortality *in utero* observed in the population where the first patients were identified (105). Intriguingly, the other forms of SCID become clinically evident only during the post-natal life, when the protection of the newborn transferred from the mother immune system declines. This observation, suggests that other causes different from immunodeficiency, are responsible for the high rate of mortality *in utero* and led to consider the Nude/SCID mutation and anencephaly causally related. Of note, in a recent study, the mouse Foxn1 gene was found to be expressed also in epithelial cells of the developing choroids plexus, a structure filling the lateral, third and fourth ventricles of the embryonic brain (105). Moreover abnormality in the development of corpus callosum were also found in another FOXN1 mutated fetus even in the absence of anencephaly, indicating that the transcription factor may play a role as a co-factor in the brain ontogeny (105).

Altogether these findings suggest that FOXN1 may also be implicated as co-factor in the development of vital systems required for a proper fetus development, thus explaining the mortality in the first trimester in fetuses carrying the genetic alterations, which is not justified by the SCID *per se*.

## FOXN1 Mutation Prevents the Pre-Natal T Cell Development in Humans

It is now clear that FOXN1 acts as a transcription factor implicated in the differentiation of thymic and skin epithelial cells, even though many of its molecular targets still remain to be discovered. Most of the knowledge so far available has been achieved in humans in the post-natal life, while little is known about FOXN1 role during the pre-natal life.

Of note, other FOX family members, including *Foxq1* and *Foxm1b*, are important during embryogenesis, being involved in a variety of biological processes (122). Approximately 50% of *Foxq1*^−/−^ murine embryos die *in utero*, thus suggesting the requirement of this gene during embryogenesis (123). Similarly, *Foxm1b* is important during liver regeneration (124).

Studies on thymus organogenesis revealed that *Foxn1* is expressed in all TECs during fetal stages. Of note, *Foxn1*^−/−^ mice showed undifferentiated TECs responsible for a blockage of thymopoiesis and severe immunodeficiency (125). Recently, the identification of a human *FOXN1*^−/−^ fetus gave the unique opportunity to study in humans the T cell development *in utero*, in the absence of a functional thymus. Vigliano et al. documented a total blockage of the CD4^+^ T cell maturation and a severe impairment of CD8^+^ cells, with an apparent bias toward TCRγδ^+^ cells (112). In this case in the congenital absence of the thymus was due to R255X missense mutation in the *FOXN1* gene. In particular, it has been reported that in the absence of FOXN1 a few not functional CD8^+^ cells, mostly bearing TCRγδ in the absence of CD3, presumably of extra-thymic origin could develop in both humans and mice (126–128). Further analysis of the fetal RNA, performed to evaluate the variable-domain β-chain (Vβ) families’ usage among T lymphocytes, revealed that the generation of TCR diversity occurred at some extent in the *FOXN1*^−/−^ fetus, but was abnormal. Thus, these data provided a further evidence of the crucial role for FOXN1 in the early pre-natal stages of T cell development and not in the B and NK-cell differentiation, these populations being normally present in the Nude/SCID fetus (112). A similar impairment of the T cell differentiation with a selective blockage of CD4 differentiation but not of CD8, was detected in murine models characterized by the absence of the nuclear high-mobility group (HMG) box protein TOX (107).

The identification of a limited number of CD8^+^ cells bearing the TCRγδ suggests that this cell population may develop at extra-thymic sites in a *FOXN1*-independent manner, even though they are unable to sustain a productive immune response into the periphery. Indeed, evidence exists indicating that T cells may also differentiate at extra-thymic sites, as intestine and liver (129–133). Of note, the majority of thymus-derived T lymphocytes bears the αβ chains of TCR and a few of them express the γδ heterodimer (134), while the T cell pool developed outside the thymus is characterized by a higher proportion of TCRγδ^+^ T cells expressing the CD8αα homodimer, instead of the CD8αβ (135, 136). Moreover, also DN T cells (CD3^+^CD4^–^CD8^–^) and lymphocytes expressing CD7 and CD2 in the absence of CD3 (CD2^+^CD3^–^CD7^+^) are generally considered of extra-thymic origin (135–137).

In spite of the well documented knowledge on the role of the primary lymphoid organ to foster T cell development, some still unsolved issues in human athymic conditions indicate that an in-depth information of the overall process is still to be achieved and, in particular, the involvement of different tissues in T cell ontogeny must be definitively clarified. Since FOXN1 is selectively expressed in the thymus and skin, one possibility to explain the presence of the few non-functional CD8^+^TCRγδ^+^ cells in Nude/SCID fetus is that skin epithelial cells could play a partial role in T cell ontogeny, as already shown in *in vitro* models (138, 139).

## Thymus Transplantation: A Promising Treatment to Athymic Disorders

*Forkhead-box n1* deficiency is a very rare immunodeficiency with unfortunately poor chance of curative treatments. Recently, thymus transplantation has emerged as a promising treatment for children affected with congenital athymia (140–143), as that observed in complete DiGeorge anomaly and in *FOXN1* deficiency. Conceptually, the thymus transplant seems to be in principle the more appropriate therapeutic strategy, taking into account that bone marrow transplantation performed in one child with *FOXN1* deficiency, failed to induce a long-term sustained immune reconstitution. In particular, in this patient no reconstitution of the naïve T cell pool was observed (144).

Thymus transplantation has been first used in children affected with complete DiGeorge anomaly, with excellent clinical and immunologic results (141). In order to achieve immune reconstitution, cultured post-natal allogeneic thymus tissue slices were transplanted into the quadriceps muscles of the athymic host (145). The migration of host bone marrow stem cells to the donor graft allow them to develop into naive T cells, which then emigrate out of the engrafted thymic tissue into the peripheral blood. Thymopoiesis is observed in biopsies of the transplanted thymus within 2 months of transplantation (140) and naive T cells are detected in the peripheral blood approximately 3–5 months after transplantation (146, 147). Taking advantage from this previous experience, a few years ago an allogeneic thymus transplantation has been used for the first time in two unrelated infants with Nude/SCID phenotype due to a deficiency of the transcription factor FOXN1 (111). The clinical phenotype of the two subjects was characterized by the absence of naïve T cells, total alopecia, nail dystrophy, and severe infections, as disseminated *Bacillus* Calmette–Guérin in subject 1 and severe respiratory infections in subject 2. Molecular analysis, performed to confirm the clinical suspect of the Nude/SCID phenotype, revealed the presence of a homozygous R255X mutation in the *FOXN1* gene in subject 1, the same of that previously described (107), and a homozygous R320W novel missense mutation in the subject 2. Moreover, subject 1 showed, like a small percentage of complete DiGeorge patients, referred as atypical complete DiGeorge, circulating oligoclonal T cells of non-maternal origins, which were predominantly double-negative T cells, and a T cell proliferative response to PHA within the normal range. Because of that, before thymus transplantation subject 1 have required immunosuppression regimen to prevent graft rejection. Differently, immunosuppression was not used for the subject 2, who had, like typical complete DiGeorge patients, very few T cells (141, 146).

Results obtained with thymus transplantation were encouraging in both FOXN1-deficient subjects, and led to a full T and B cell reconstitution and functional rescue. Indeed, both subjects developed naïve T cells, diverse TCR repertoires and an *in vitro* proliferative T cell responses against different antigens. Eventually they reached normal serum Ig levels with generation of protective antibody specific titers. Of note, HLA matching for class I and II did not seem to interfere with T cell counts after thymus transplantation, being subject 2 transplanted without any HLA matches. However, CD8^+^ T cell number, although apparently functional, was disproportionally low compared to CD4^+^ T cells (111). A poor CD8 recovery has also been described in complete DiGeorge patients, who underwent HLA-mismatched thymic transplantation (141, 148). Possible explanations are that the phenomenon is related to the HLA mismatch between host hematopoietic precursors and allograft thymic epithelia or to alterations in the thymic graft due to transplantation procedures.

Functionality of the thymic allograft has been assessed for the first time through signal joint (sj) and DβJβ T cell receptor rearrangement excision circle (TREC) analyses (109). The sj/βTREC represents a ratio between early and late products of TCR rearrangements, which directly correlate with thymic output and provide an indirect measurement of thymocyte division-rate (149– 151). The sj/βTREC ratio quantification, conducted in subject 1 with R255X mutation, was very low during the peri-transplant period and comparable to those observed in healthy children at 2.5 years post-transplant. Of note, 4 years post-transplantation a decrease of sj/βTREC ratio associated with a reduction in sjTREC levels and in the number of naïve cells were found, suggesting the decline in thymic allograft output (109). This decline might be due to the reduced longevity of the thymus allograft or to peripheral homeostasis of the T cell pool maintenance following its replenishment. Overall, the thymus transplantation seems to be a promising curative strategy for subjects with athymia due to *FOXN1* deficiency or complete DiGeorge syndrome in the perspective of long-term clinical benefit.

## Conclusion

The integrity of the thymic epithelial architecture allows the growth, the differentiation, and TCR repertoire selection of immature T cells, thus originating fully mature and functional T cells. Of note, the failure to generate or to maintain the proper 3D thymic architecture leads to severe immunodeficiency or autoimmunity. The unique function of the thymus in the establishment/maintenance of the T cell pool is related not only to the peculiar 3D structure, but also to the specialized functions of the thymic stroma. Indeed, lympho-stromal interactions within the multicellular thymic microenvironment play a crucial role in the regulation of the T cell development. Moreover, these interactions are based on a bilateral crosstalk between stromal cells and traveling thymocytes, which, in turn, are able to provide important signals for the TECs differentiation.

Thymus organogenesis and T cell development are sophisticated biological processes, which require the activation of a wide panel of genes. There is evidence that the master regulator of the thymus development is the *Foxn1* gene, since it is required at multiple intermediate stages of the TE-lineage specification either in the fetal and adult thymus, through the direct or indirect regulation of genes involved in the thymus development and function. These genes include *Pax1*, *Hoxa3*, *CCL25*, *Dll4*, *p63*.

Studies on the animal and human model of the Nude/SCID phenotype have provided an enormous contribution in identifying the crucial role of *Foxn1* to drive the thymus development, even though many issues regarding the transcriptional regulation of the TECs specification and homeostasis still remain to be solved. The development *in vitro* of cellular models of TEC lineage differentiation, by using the technology of nuclear reprograming, will be certainly useful to better characterize the discrete stages of the TECs differentiation and the molecular mechanism involved in the process.

Eventually, the *in vitro* re-build of a thymic environment capable to reproduce tissue features of primary lymphoid organs (139, 152) could be a promising and valuable tool for the treatment of congenital athymia, including *FOXN1* deficiency, along with the thymus transplantation, which is emerged as a potential treatment for these disorders.

## Conflict of Interest Statement

The authors declare that the research was conducted in the absence of any commercial or financial relationships that could be construed as a potential conflict of interest.
